# The Objective Assessment of Cough Frequency in Bronchiectasis

**DOI:** 10.1007/s00408-017-0038-x

**Published:** 2017-07-13

**Authors:** Arietta Spinou, Kai K. Lee, Aish Sinha, Caroline Elston, Michael R. Loebinger, Robert Wilson, Kian Fan Chung, Nadia Yousaf, Ian D. Pavord, Sergio Matos, Rachel Garrod, Surinder S. Birring

**Affiliations:** 1 Division of Asthma, Allergy and Lung Biology, King’s College London, Denmark Hill, London, SE5 9RS UK; 2 Department of Respiratory Medicine, King’s College Hospital, Denmark Hill, London, UK; 3 Host Defence Unit, Royal Brompton Hospital, London, UK; 4 NIHR Respiratory Biomedical Research Unit, Royal Brompton NHS Foundation Trust and Imperial College London, London, UK; 5 Department of Medicine, The Royal Marsden Foundation Trust, London, UK; 6 Department of Respiratory Medicine, Nuffield Department of Medicine, University of Oxford, Oxford, UK; 7 Institute of Electronics and Telematics Engineering (IEETA), University of Aveiro, Aveiro, Portugal; 8 King’s College London, Therapies, Denmark Hill Campus, London, UK

**Keywords:** Cough frequency, Bronchiectasis, Leicester cough monitor, Visual analogue scale, Bronchiectasis Health Questionnaire

## Abstract

**Introduction:**

Cough in bronchiectasis is associated with significant impairment in health status. This study aimed to quantify cough frequency objectively with a cough monitor and investigate its relationship with health status. A secondary aim was to identify clinical predictors of cough frequency.

**Methods:**

Fifty-four patients with bronchiectasis were compared with thirty-five healthy controls. Objective 24-h cough, health status (cough-specific: Leicester Cough Questionnaire LCQ and bronchiectasis specific: Bronchiectasis Health Questionnaire BHQ), cough severity and lung function were measured. The clinical predictors of cough frequency in bronchiectasis were determined in a multivariate analysis.

**Results:**

Objective cough frequency was significantly raised in patients with bronchiectasis compared to healthy controls [geometric mean (standard deviation)] 184.5 (4.0) vs. 20.6 (3.2) coughs/24-h; mean fold-difference (95% confidence interval) 8.9 (5.2, 15.2); *p* < 0.001 and they had impaired health status. There was a significant correlation between objective cough frequency and subjective measures; LCQ *r* = −0.52 and BHQ *r* = −0.62, both *p* < 0.001. Sputum production, exacerbations (between past 2 weeks to 12 months) and age were significantly associated with objective cough frequency in multivariate analysis, explaining 52% of the variance (*p* < 0.001). There was no statistically significant association between cough frequency and lung function.

**Conclusions:**

Cough is a common and significant symptom in patients with bronchiectasis. Sputum production, exacerbations and age, but not lung function, were independent predictors of cough frequency. Ambulatory objective cough monitoring provides novel insights and should be further investigated as an outcome measure in bronchiectasis.

**Electronic supplementary material:**

The online version of this article (doi:10.1007/s00408-017-0038-x) contains supplementary material, which is available to authorized users.

## Introduction

Bronchiectasis is a chronic condition that is characterised by dilated and often thick walled bronchi [1, 2]. Cough is a predominant symptom of bronchiectasis and is worse during exacerbations [3]. Cough in bronchiectasis is associated with significant impairment in health-related quality of life (HRQOL) [4]. Adverse symptoms associated with cough include incontinence, syncope, chest pain and social embarrassment [5, 6].

The development of ambulatory cough monitoring devices has facilitated the objective assessment of cough frequency [7, 8]. Recent studies in chronic respiratory disorders such as chronic obstructive pulmonary disease (COPD) and sarcoidosis have reported that patients cough frequently, and this is associated with impaired HRQOL [8, 9]. Objective cough frequency is also raised in tuberculosis and has been linked to its transmission [10–12]. An advantage of objective cough monitoring over subjective outcome measures is that it is not susceptible to the perception of cough severity and reflects actual coughing. There is a paucity of studies that have investigated objective cough frequency in bronchiectasis. The aim of this pilot study was to investigate cough frequency objectively with 24-h cough monitoring and its association with self-reported cough severity and HRQOL. We also investigated clinical predictors of objective cough frequency.

## Methods

### Subjects and Clinical Characterisation

Consecutive adult patients with bronchiectasis were recruited prospectively from secondary care (King’s College Hospital) and tertiary care (Royal Brompton Hospital) specialist clinics from November 2012 to August 2014. The diagnosis of bronchiectasis was based on clinical characteristics, computerised tomography (CT) scans and consistent with the British Thoracic Society guidelines [1]. Exclusion criteria were cystic fibrosis, upper respiratory tract infection or exacerbation of bronchiectasis within the past two weeks, current smokers, presence of other co-existing respiratory conditions and angiotensin-converting enzyme inhibitor medication. The presence of cough was not an inclusion criterion. Demographics and clinical characteristics were recorded with a structured questionnaire. Sputum colonisation status was recorded using the clinical records of the most recent sputum analyses. Sputum bacterial colonisation status was defined as at least 2 positive cultures, assessed a minimum 3 months apart and within one year [13]. The objective cough frequency data from 35 healthy controls (restricted to age range of patients with bronchiectasis) from a previous study were used for comparison [14]. All healthy subjects had normal spirometric values and were non-smokers (never or ex-smokers <10 pack year history) and asymptomatic. All subjects gave informed written consent and the study was approved by the local research ethics committee (NRES Committee London - Queen Square, 12/LO/1437).

### Cough Frequency Monitoring

Cough frequency was recorded with the Leicester Cough Monitor (LCM). The LCM is a validated ambulatory cough monitor that consists of a portable MP3 sound recorder and free-field microphone, worn for 24 h in the patient’s own environment [7, 15, 16]. The sound files were uploaded onto a computer for automated analysis using customised cough detection software described previously [17]. Coughs were detected as single events whether occurring in isolation or bouts. Patients were asked to record the times of physiotherapy airway clearance in a diary. The cough monitor methodology used in this study was similar to that of healthy controls [14].

### Subjective Assessment of Cough, Sputum and Health Status

The Leicester Cough Questionnaire (LCQ) was used to assess the impact of cough on the patients’ HRQOL and the cough-specific visual analogue scale (VAS, 0–100 mm) was used to assess cough severity. The LCQ is a validated cough-specific HRQOL questionnaire for adults that has been validated in bronchiectasis [4, 18]. It has 19 cough-specific questions that are divided into three domains (physical, psychological and social) and a 7-point Likert response scale. Scores for each domain range from 1 to 7 and total score range is 3–21, with a higher score indicating a better HRQOL. Sputum production was assessed with item 2 from the St. Georges Respiratory Questionnaire (SGRQ): “Over the past 4 weeks, I have brought up phlegm (sputum): not at all/only with respiratory infections vs. a few days a month, several days a week or most days a week” [8, 19, 20]. Patients were also asked to complete VAS scales for severity of breathlessness and sputum production. HRQOL was assessed with a respiratory questionnaire, SGRQ, and with a novel, validated, disease specific HRQOL questionnaire, Bronchiectasis Health Questionnaire (BHQ) [21–23]. The BHQ has 10 items and patients respond on a 7-point Likert scale. This questionnaire generates a single total score, range 0–100, with higher scores indicating better health status. Self-reported frequency of antibiotic use for respiratory tract infections was used as an indicator of the frequency of exacerbations in the previous 12 months (excluding past 2 weeks). This was assessed with item 10 of the BHQ: “In the last 12 months, I have taken antibiotics for a chest infection” (0, 1, 2, 3, 4, 5, >5 times) [21–23].

### Lung function

Spirometry was measured clinically in accordance with international guidelines [24].

### Protocol

All patients completed the questionnaires and were set up for 24-h cough monitoring on the first day. They returned the cough monitor the next day at the same time.

### Analysis

Data were analysed using Prism^®^ Version 5.0 for Windows (GraphPad Software; San Diego, California, USA) and SPSS^®^ Statistics Version 20.0 for Windows (IBM, SPSS Inc; Chicago, Illinois, USA). The distribution of data was assessed using the D’ Agostino and Pearson omnibus test. Parametric data were expressed as mean (standard deviation, SD), whereas non-parametric data were expressed as median (interquartile range, IQR). The cough frequency and count data were logarithmic-transformed and presented as geometric mean (log SD). Parametrically distributed data were analysed with independent sample t tests to compare sample means, whereas comparison of non-parametric data was carried out using the Mann–Whitney *U* test. All analyses included subjects with and without cough unless otherwise stated. The normal ranges for females and males have previously been reported; females <5 coughs per hour and males <2 coughs per hour [14]. Correlations between variables were analysed with the Pearson’s correlation coefficient (*r*) for parametric data and the Spearman’s correlation coefficient (*ρ*) for non-parametric data. A p value of less than 0.05 was considered statistically significant. Predictors of objective cough frequency were assessed in patients with bronchiectasis using Pearson’s and Spearman’s correlations and general linear models. All subjects with bronchiectasis were included in the univariate and multivariate analyses.

## Results

### Subject Characteristics

Fifty-seven patients with bronchiectasis were recruited for this study. The characteristics of patients are shown in Table 1. Two patients were excluded due to cough recording duration less than 24-h and one participant was unable to return the cough monitor device. Fifty-four patients completed a 24-h cough monitoring. The most common identified cause of bronchiectasis was post-infection (30%). Forty-six percent of patients had idiopathic bronchiectasis. Bacterial sputum colonisation (any micro-organism) was present in 22 (41%) patients and *Pseudomonas aeruginosa* colonisation in 14 (26%). The proportion of patients that reported taking antibiotic courses for acute respiratory infections in the past 12 months were as follows: 14% no courses, 10% one course, 12% two courses, 21% three courses, 8% four courses, 12% five courses and 23% of patients more than five courses. Twenty-one percent of patients reported having an acute hospital admission for their bronchiectasis in the previous 12 months.

**Table 1:** Demographic and clinical characteristics of the study participants

	Bronchiectasis (*n* = 54)	Healthy (*n* = 35)
Female, *n* (%)	37 (69)	18 (51)
Age, years^#^	60.5 (15.0)	49.8 (13.9)*
Body mass index, kg/m^2^	23.0 (20.0, 28.0)^#^	25.1 (22.6, 29.0)^#^
Smoking status, *n* (%)		
Never smoker	42 (78)	35 (100)
Ex-smoker (>10 pack year)	12 (22)	0 (0)
Current smoker	0 (0)	0 (0)
Spirometry		
FEV_1_ % predicted	70.7 (26.2)	93.4 (25.8)*
FEV_1_/FVC^#^	65.2 (13.7)	79.68 (5.2)*
PSA colonisation	14 (25.6)	–
Symptoms severity		
Sputum VAS	28.0 (15.0, 54.0)^#^	na
Dyspnoea VAS	26.5 (12.3, 61.8)^#^	na
SGRQ		
Symptoms	64.0 (19.1)	na
Activities	50.1 (28.1)	na
Impact	32.5 (21.1)	na
Total	40.1 (20.1)	na
BHQ	60.6 (11.7)	na
Aetiology, *n* (%)		
Idiopathic	25 (46.3)	na
Post infectious	16 (29.6)	na
Immunodeficiency	5 (9.3)	na
Other	6 (11.1)	na
ABPA	2 (3.7)	

Data presented as mean (standard deviation, SD), *n* (%), or medians (interquartile range, IQR). Healthy individuals’ data are historical [12]

*ABPA* allergic bronchopulmonary aspergillosis, *BHQ* Bronchiectasis Health Questionnaire, *FEV*_*1*_ forced expiratory volume in the first second, *FVC* forced vital capacity, *PSA P. aeruginosa, SGRQ* St George’s Respiratory Questionnaire, *VAS* visual analogue scale, *na* not applicable

^#^Data presented as medians (IQR)

* Indicates significant differences between the groups, *p* < 0.05

### Cough Frequency Monitoring

#### Bronchiectasis vs. Healthy Controls

**Fig. 1:**
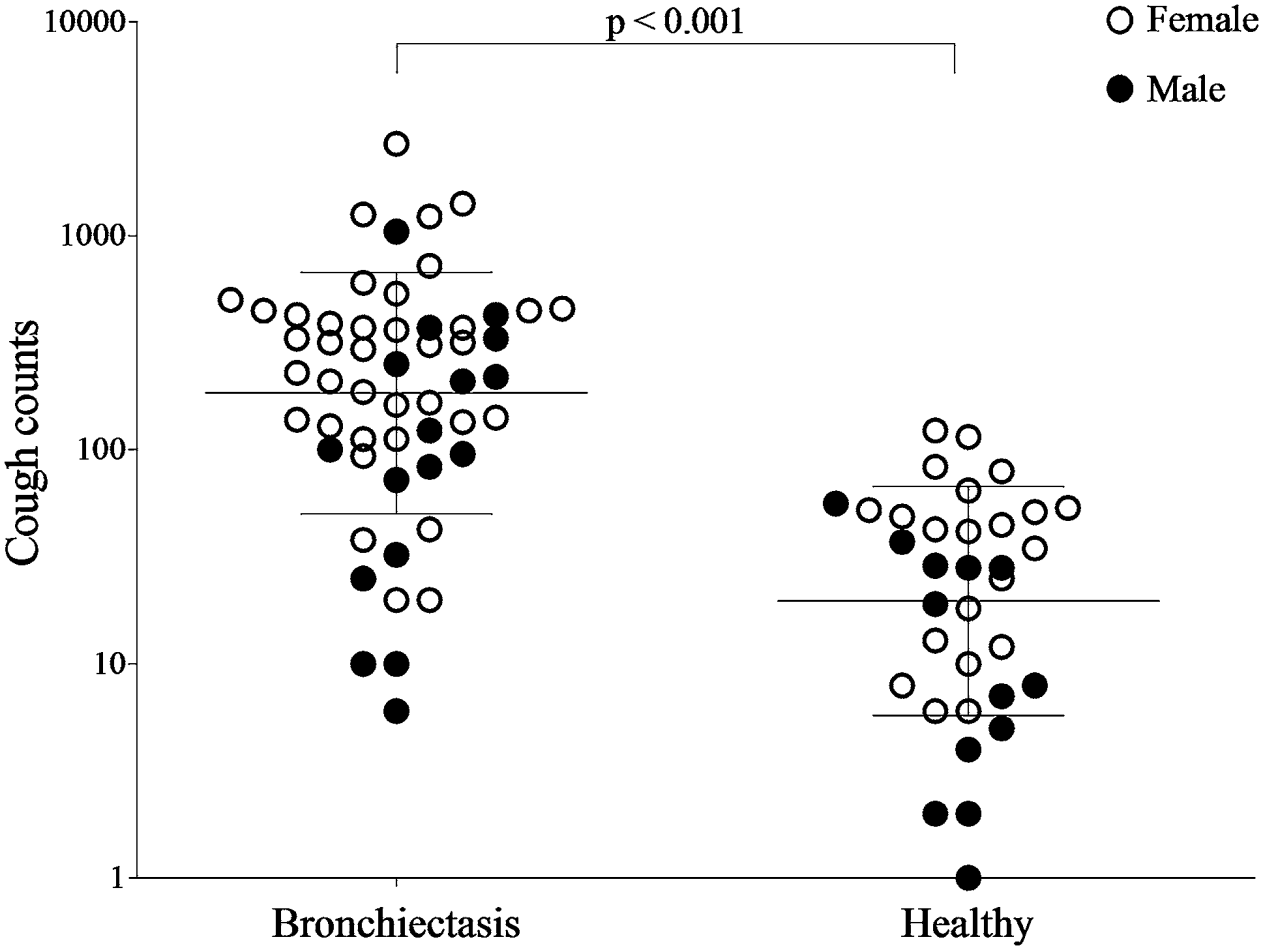
Comparison of 24-h cough counts between patients with bronchiectasis (*n* = 54) and healthy individuals (*n* = 35). Data presented as geometric mean (standard deviation, SD). *Open circles* represent female participants. *Closed circles* represent male participants. Healthy individuals’ data are historical [11]. Objective cough counts per 24 h were measured using the Leicester Cough Monitor. There was a statistically significant difference in cough counts per 24 h between female and male participants (*p* = 0.006)

Twenty-four hour cough counts were significantly higher in patients with bronchiectasis compared to healthy individuals; geometric mean (logSD) 184.5 (0.6) coughs vs. 20.6 (0.5) coughs respectively, mean fold-difference (95% confidence intervals, CI) 8.9 (5.2, 15.2), *p* < 0.001 (Fig. 1). Forty-one (84%) patients with bronchiectasis had abnormally raised cough frequency based on a previously published normal range (females <5 coughs per hour and males <2 coughs per hour) [7, 14].

#### Bronchiectasis

**Fig. 2:**
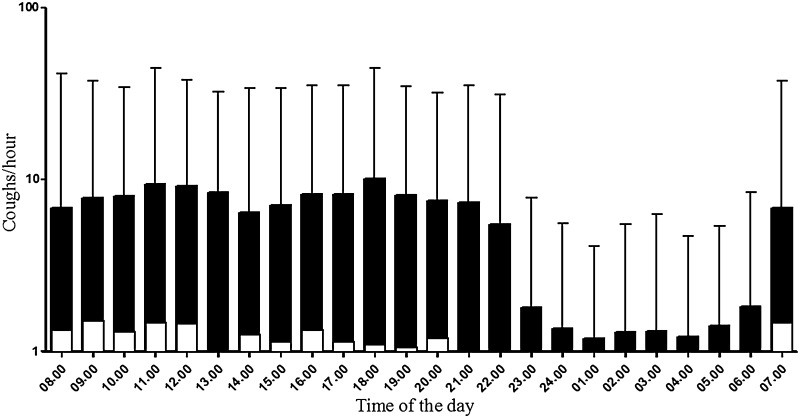
Number of coughs per hour during the 24-h cough frequency recording in bronchiectasis (*n* = 54) and healthy participants (*n* = 35). Data presented as geometric mean (standard deviation, SD) Objective cough counts per 24 h were measured using the Leicester Cough Monitor. Healthy participants represented in white, bronchiectasis participants in *black*

Daytime (awake) cough counts were significantly greater than night-time (asleep) in patients with bronchiectasis; geometric mean (logSD) of daytime coughs 164.4 (0.6) vs. night-time coughs 14.2 (0.8), mean fold-difference (95% CI) 11.6 (8.2, 16.2), p < 0.001 (Fig. 2). The frequency and impact of cough are presented in Table 2. Female patients had significantly higher cough counts per 24 h compared to male patients; geometric mean (logSD): 254.7 (0.5) coughs vs. 91.4 (0.6) coughs, respectively, mean fold-difference (95% CI) 2.8 (1.4, 5.7); *p* = 0.006, Fig. 1. There was no significant difference in 24-h cough counts between patients with idiopathic bronchiectasis and identified cause; geometric mean (logSD) 240.5 (0.1) coughs vs. 146.6 (0.1), respectively, geometric mean fold-difference (95% CI) 1.6 (0.1, 3.3), *p* = 0.165.

**Table 2:** Objective and subjective assessments of cough in patients with bronchiectasis (*n* = 54) and healthy individuals (*n* = 35)

Cough outcome measure	Bronchiectasis	Healthy
Cough severity, median (IQR)		
VAS cough	31.0 (15.8, 67.0)	na
Impact on health status, median (IQR)		
LCQ Physical	4.5 (3.2, 5.8)	na
LCQ Psychological	5.3 (3.4, 6.3)	na
LCQ Social	5.3 (4.3, 6.3)	na
LCQ Total	15.3 (10.4, 18.5)	na
Objective cough counts, geo mean (logSD)		
24-h cough counts, n	184.5 (0.6)	20.6 (0.5)**
Daytime cough counts, n	164.4 (0.6)	13.4 (0.5)**
Night-time cough counts, n	14.2 (0.8)	6.5 (0.5)*

Data presented as median (interquartile range, IQR) or geometric mean (logarithmic standard deviation, logSD)

*LCQ* Leicester Cough Questionnaire, *geo mean* geometric mean, *na* not applicable

* *p* < 0.05, ** *p* < 0.001

The impact of intentional coughing during home, self-directed, airway clearance physiotherapy was assessed. Twenty-eight patients with bronchiectasis reported performing at least one self-physiotherapy session for airway clearance during 24-h cough monitoring and twelve reported performing ≥2 sessions. The median (IQR) self-reported duration for each session was 20 (10, 50) minutes. There was no significant difference between 24-h cough counts in patients who reported doing airway clearance vs. those who did not (*n* = 26); geometric mean (logSD) 146.6 (0.5) vs. 139.6 (0.6) coughs, respectively, mean fold-difference (95% CI) 1.7 (0.8, 3.4), *p* = 0.131. Geometric mean (logSD) cough counts during the hour in which airway clearance physiotherapy was performed was higher than daytime cough frequency, but this was not statistically significant; 21.7 (0.6) vs. 14.5 (0.5) coughs per hour, respectively, geometric mean fold-difference (95% CI) 1.5 (0.8, 2.8), *p* = 0.203. There was no significant difference between cough counts in the 2-h preceding and 2-h following airway clearance; geometric mean (logSD) 33.6 (0.5) vs. 31.0 (0.5) coughs, respectively, mean fold-difference (95% CI) 1.1 (0.7, 1.7), *p* = 0.728. It should be noted that the study sample size was small for these sub-analyses and potentially underpowered to detect differences.

#### Cough Frequency of Sputum Producers (Bronchiectasis)

Thirty-six (66.7%) patients reported sputum production, median (IQR) VAS for sputum severity was 45 (16, 64) mm. The 24-h cough counts was significantly higher in sputum producers compared to non-producers; geometric mean (logSD) 281.2 (0.8) vs. 49.8 (0.2), respectively, mean fold-difference (95% CI) 5.6 (1.6, 20.0), *p* = 0.013. The cough severity VAS and cough-HRQOL (LCQ total) scores in sputum producers compared to non-producers were: median (IQR) VAS 33 (21, 68) vs. 28 (11, 52) mm, respectively, *p* = 0.385 and median (IQR) LCQ total score 15.2 (10.7, 18.6) vs. 17.5 (14.6, 19.0), respectively, *p* = 0.465.

### The Relationship Between Cough Frequency and Subjective Assessments of Cough, Sputum and HRQOL (Bronchiectasis)

**Fig. 3:**
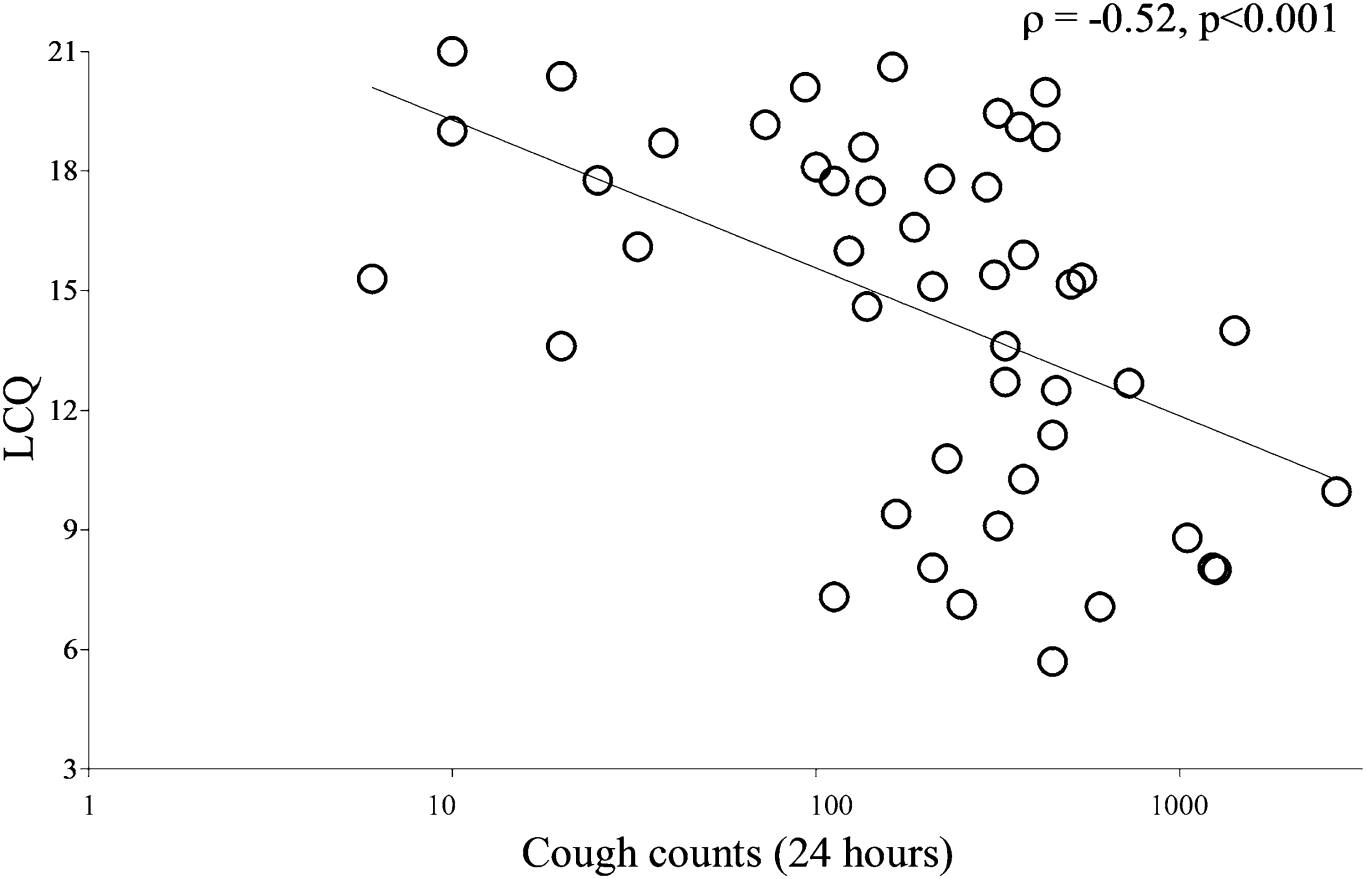
Relationship between 24-h cough counts and cough-related health status, using the Leicester Cough Questionnaire (LCQ). *ρ*: Spearman’s correlation coefficient. Health status was measured using the Leicester Cough Questionnaire. Objective cough counts per 24 h were measured using the Leicester Cough Monitor

**Fig. 4:**
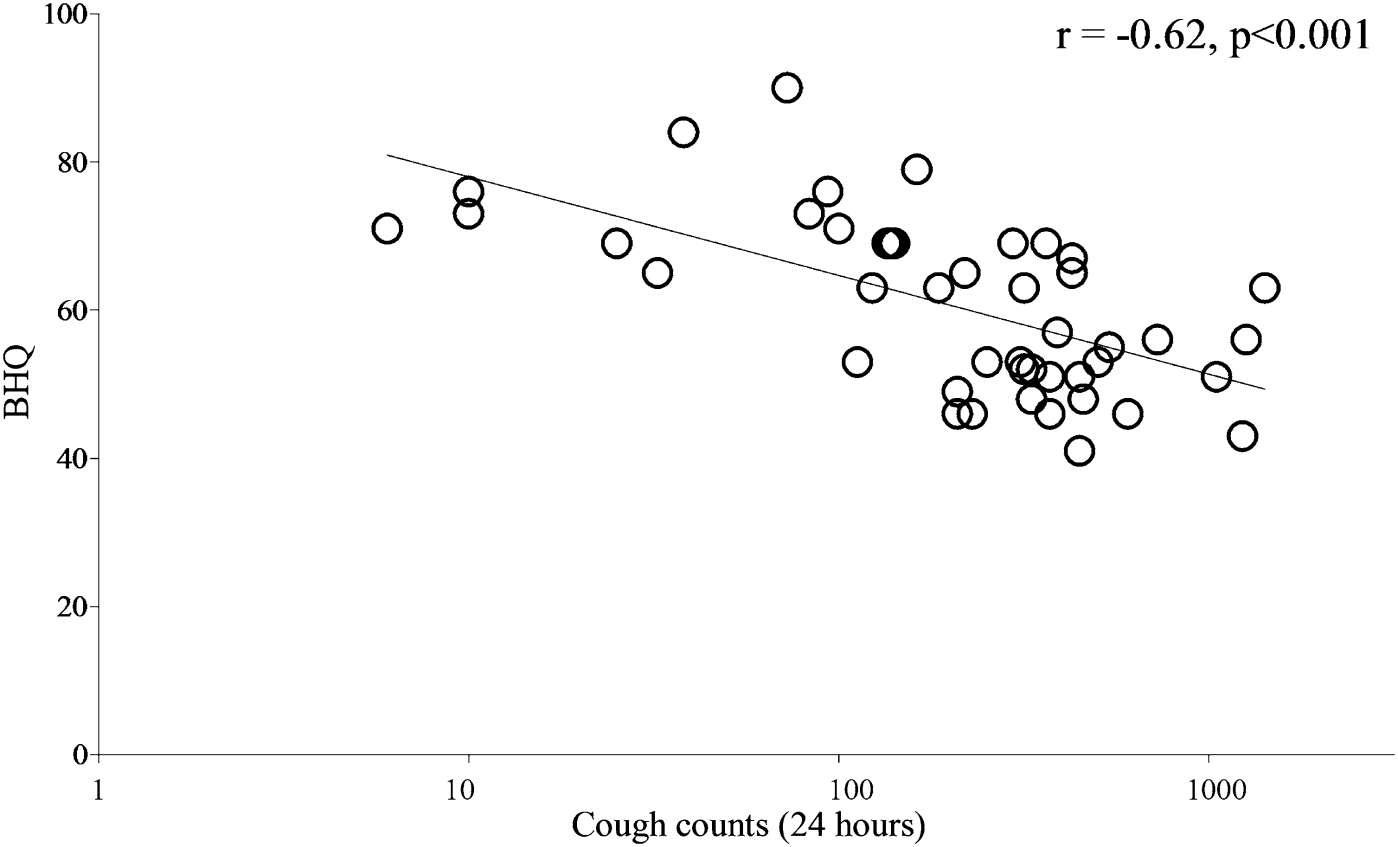
Relationship between 24-h cough counts and health status, using the Bronchiectasis Health Questionnaire (BHQ). *r*: Pearson’s correlation coefficient. Health status was measured using the Bronchiectasis Health Questionnaire. Objective cough counts per 24 h were measured using the Leicester Cough Monitor

Patients with bronchiectasis reported a moderate cough severity on VAS; median (IQR) 31 (16, 67) mm. Cough impacted all domains of HRQOL, see Table 2. There was a significant association between 24-h cough counts and cough-specific HRQOL (LCQ total score *ρ* = −0.52, *p* < 0.001; Fig. 3); cough severity VAS (*ρ* = 0.54, *p* < 0.001; online resource 1); and sputum VAS (*ρ* = 0.50, *p* < 0.001; online resource 2). There was stronger correlation between 24-h cough counts and bronchiectasis HRQOL assessed with BHQ (*r* = −0.62, *p* < 0.001; Fig. 4) than SGRQ (total score *r* = 0.32, *p* = 0.031; Table 3 and online resource 3).

**Table 3:** The association between 24-h cough counts and health status in bronchiectasis (*n* = 54)

Questionnaire	Correlation coefficient	*p* value
SGRQ		
Symptoms	0.320	0.025
Activity	0.210	0.161
Impact	0.352	0.017
Total	0.323	0.031
LCQ^#^		
Physical	−0.556	<0.001
Psychological	−0.475	0.001
Social	−0.487	<0.001
Total	−0.520	<0.001
BHQ		
Total	−0.616	<0.001

Data presented as Pearson’s correlation coefficient (*r*), unless otherwise stated

*BHQ* Bronchiectasis Health Questionnaire, *LCQ* Leicester Cough Questionnaire, *SGRQ* St George’s Respiratory Questionnaire

^#^ Correlation coefficient Spearman’s *ρ*

### Clinical Predictors of Objective Cough Frequency (Bronchiectasis)

Table 4 summarises the relationship between patient characteristics and objective cough frequency in the univariate and multivariate models. In the univariate analyses, gender, reported sputum production and number of courses of antibiotics for respiratory infections in past year correlated significantly with cough frequency. There was a trend towards significance for age and sputum colonisation with *P. aeruginosa*. There was no significant relationship between cough frequency and lung function (Table 4, online resource 4 and online resource 5), smoking status and aetiology of bronchiectasis. Significant influences on objective cough frequency were further explored in a multivariate model, including five of the most statistically significant/near significance variables in univariate analysis (gender, age, reported sputum production, *P. aeruginosa* colonisation and antibiotics for respiratory infection frequency). Incorporating gender, age, reported sputum production, *P. aeruginosa* colonisation and antibiotic frequency into the model explained 52% of the variance in cough frequency (*p* < 0.001). Age, reported sputum production and frequency of antibiotics for respiratory infection remained significant predictors of cough frequency within the multivariate model (Table 4). Gender (*p* = 0.07) and sputum *P. aeruginosa* colonisation (*p* = 0.06) approached statistical significance (Table 4).

**Table 4:** Predictors of objective cough frequency in patients with bronchiectasis (*n* = 54)

Predictors of cough frequency	Correlation coefficient or variance	*p* value
Univariate analyses		
Age	*r* = 0.229	0.096
Sex		0.006*
Body mass index	*r* = −0.082	0.594
Spirometry		
FEV_1_ % predicted	*r* = −0.139	0.362
FVC % predicted	*r* = −0.131	0.392
FEV_1_/FVC	*r* = 0.026	0.857
Aetiology of bronchiectasis		0.276
Smoking status (never/ex)		0.268
Sputum production		**0.013**
Sputum microbiology		
*Pseudomonas* sputum colonisation (yes/no)		0.091
Exacerbation frequency (past 1 year)	*ρ* = −0.327	**0.029**
Multivariate analyses		
Model	*R* ^2^ = 52.0%	**<0.001**
Sex	−0.230	0.077
Age	0.279	**0.016**
*Pseudomonas* sputum colonisation	0.207	0.066
Sputum production	0.448	**<0.001**
Exacerbation frequency	−0.284	**0.028**

Bold values indicate *p* values <0.05

Data represent the ability of variables observed for predicting cough frequency. Multivariate analysis was carried out using variables that had statistically significant or nearly significant association with cough frequency; sex, age, self-reported sputum production, sputum *P. aeruginosa* colonisation and exacerbation frequency (past one year). Self-reported sputum production was assessed by item 2 of the St George’s Respiratory Questionnaire [18]. Respiratory infections were assessed by self-reported frequency of antibiotics courses for respiratory infections in the past year. *ρ* = Spearman’s correlation coefficients

*FEV*_*1*_ forced expiratory volume in the first second, *FVC* forced vital capacity, *SGRQ* St George’s Respiratory Questionnaire, *VAS* visual analogue scale

* Cough frequency greater in females compared to males

## Discussion

This is the first study to investigate 24-h objective cough frequency in patients with bronchiectasis. Cough frequency was increased compared to healthy individuals, and was associated with significant impairment in HRQOL. Age, sputum production and frequency of antibiotic use for respiratory exacerbations were independent predictors of cough frequency, explaining 52% of its variance. There was no association between cough frequency and lung function. The strongest association between objective cough frequency and patient-reported outcomes measures was with the Bronchiectasis Health Questionnaire (BHQ).

The cough frequency of bronchiectasis patients was significantly higher than in healthy individuals, comparable with that published in previous studies of chronic obstructive pulmonary disease and less than idiopathic chronic cough [8, 25]. Patients with bronchiectasis had a diurnal variation in cough frequency, being significantly higher during the day compared with the night. This is consistent with findings in patients with chronic cough and healthy individuals [6, 7, 16, 17, 26, 27]. Patients with bronchiectasis were older than healthy controls. It is unlikely that age alone was the major reason for the ninefold difference in cough frequency in patients compared to healthy controls. There was no significant relationship between cough frequency and age in univariate analysis; however, this was statistically significant in multivariate analysis. The basis for this relationship with age is not clear. There is no relationship with age reported in other chronic respiratory disorders such as idiopathic pulmonary fibrosis, sarcoidosis, COPD or healthy individuals [8, 9, 14, 28]. There was an increase in cough frequency during the hour in which patients self-reported performing airway clearance physiotherapy at home, compared to the average daytime cough frequency. This increase in cough frequency was not statistically significant and represented a small proportion of overall 24-h cough counts. There was no significant difference in cough frequency in the 2 h preceding home airway clearance compared with 2 h following this. This study wasn’t designed to investigate the impact of airway clearance therapy and this should be assessed in larger studies.

We found that age, sputum production and frequency of antibiotics for respiratory infections were significant independent predictors of objective cough frequency. Sputum production was an independent predictor of cough frequency similar to the findings reported by Sumner et al. in COPD [8]. In contrast, Sinha et al. did not find such association in sarcoidosis [9]. We did not investigate if specific characteristics of sputum were associated with cough frequency such as volume, colour and consistency; this should be investigated in future. We found a weak but statistically significant association between cough frequency and the number of courses of antibiotics in the previous one year for respiratory infections. The frequency of antibiotics was however a significant independent predictor of cough frequency in multivariate analysis. A recent study by Kapur et al. in children with bronchiectasis found that self-reported cough severity and the presence of “wet cough” were the strongest predictors for defining an exacerbation, by a considerable margin amongst a wide range of commonly used clinical makers [29]. The potential of objective cough monitoring for defining exacerbations in research studies should be explored.

There was a weak association between cough frequency and sputum colonisation with *P. aeruginosa* in univariate and multivariate analysis, which approached statistical significance. Larger studies are needed to investigate the effect of airway micro-organism colonisation on cough since our study was underpowered to investigate this. Cough reflex sensitivity has been reported to be heightened in bronchiectasis, and is associated with subjectively reported cough severity, similar to other chronic respiratory disorders such as sarcoidosis and idiopathic chronic cough [30, 31]. The sensitivity of the cough reflex may therefore be an important determinant of cough frequency. We did not study the mechanisms that may be important in determining the frequency of cough, as this was beyond the scope of this study. Future studies should investigate the association between cough frequency and cough reflex sensitivity, airway hyper-responsiveness, airway inflammation and the extent of bronchiectasis, mucus plugging and airway wall thickening using CT scan scoring systems [7]. We did not find an association between cough frequency and standard lung function parameters, and this finding is similar to those in COPD, idiopathic chronic cough and sarcoidosis [8, 9]. The assessment of cough in bronchiectasis is therefore likely to require tools other than lung function measures.

A recent systematic review and meta-analysis reported that cough is one of the most important determinants of HRQOL in bronchiectasis [32]. Our study confirms that HRQOL is significantly impaired in bronchiectasis. There was only a moderate relationship between subjective and objective assessments of cough. The poor association between subjective and objective tools is further demonstrated by our finding that despite a near fivefold difference in cough frequency between sputum producers and non-producers, the subjective assessments of cough were not statistically different between these groups. This may suggest that individuals with a dry cough are more troubled by their cough. Subjective measures assess aspects of cough different to those measured by objective instruments, and perhaps are more important to patients since they represent their perception of the condition. A number of cough outcomes are now available to assess patients with bronchiectasis. They are best used in combination to assess cough comprehensively. Among all subjective tools, the BHQ had the strongest association with objective cough frequency, followed closely by the LCQ and VAS. The weakest association was with the SGRQ. These findings highlight the importance of using disease and symptom-specific tools when assessing patients.

There are some limitations to our study. We studied a small number of subjects and this may have led to clinically relevant imbalances in variables. Therefore, our findings need to be confirmed in larger studies. We did not record treatment status of participants and this could have impacted the frequency of cough. We did not assess patients for potential causes of cough, such as laryngeal and sinus disease, gastro-oesophageal reflux and asthma. It is possible that the presence of gastro-oesophageal reflux in some patients may have influenced cough frequency since it is associated with increased frequency of exacerbations of bronchiectasis and sputum colonisation. The purpose of this study was to investigate objective cough frequency in unselected patients with bronchiectasis. Future studies should assess the relationship between cough frequency, aetiology, therapy and patho-physiological mechanisms, such as airway hyper-responsiveness, cough reflex sensitivity and airway inflammation. We did not find an association between cough, and FEV_1_ or the presence of airway colonisation with *P. aeruginosa*. These severity markers have limitations when used to assess disease severity and therefore future studies should assess disease severity with validated tools, such as the Bronchiectasis Severity Index (BSI), which was not available at the time of study, FACED and the extent of bronchiectasis on CT scanning [14, 33]. We did not record MRC breathlessness scale that is required to calculate the BSI. We also note that the tools developed to assess cough frequency have not formally been validated in bronchiectasis. Cough in a patient with bronchiectasis often sounds different to that of idiopathic cough. It is not known whether the characteristics of cough sounds in bronchiectasis affects the ability of cough monitors to detect cough compared to other cough disorders. We found a diurnal variation in cough frequency, higher frequency in females compared to males and a significant association with subjective assessments of cough, consistent with chronic dry cough disorders. Furthermore, the cough monitor was able to identify differences in cough frequency between patients reporting sputum production compared with non-producers. Cough monitors have been used in a wide range of respiratory conditions such as asthma [34], COPD [8], chronic cough [7], IPF [28], acute upper respiratory tract infection [15], sarcoidosis [9] and cystic fibrosis [35]. Future studies should investigate the performance of cough detection monitors in bronchiectasis. It is possible that some coughs detected with monitors where those of surrounding subjects in the patients’ environment but we have recently reported that the Leicester Cough Monitor is able to discriminate patient from environmental coughs (cough from subjects nearby) [36].

The findings of this study suggest that cough is common in patients with bronchiectasis, and is associated with significant impairment in HRQOL. Our data also suggest that it is feasible to assess cough objectively with 24-cough frequency monitors. Cough frequency was significantly raised in patients with bronchiectasis compared to healthy individuals. Age, sputum production and frequency of antibiotic use for respiratory infections were independent predictors of cough frequency. Objective cough frequency monitoring should be investigated further as an outcome measure in bronchiectasis.

## Electronic supplementary material

Below is the link to the electronic supplementary material.

Supplementary material 1 (DOCX 11 kb)

Supplementary material 2 (TIFF 153 kb)

Supplementary material 3 (TIFF 150 kb)

Supplementary material 4 (TIFF 161 kb)

Supplementary material 5 (TIFF 146 kb)

Supplementary material 6 (TIFF 138 kb)

## Abbreviations

LCM: Leicester cough monitor
LCQ: Leicester Cough Questionnaire
HRQOL: Health-related quality of life
VAS: Visual analogue scale
FEV_1_: Forced expiratory volume in 1 s
FVC: Forced vital capacity
SD: Standard deviation
COPD: Chronic obstructive pulmonary disease
IPF: Idiopathic pulmonary fibrosis
SGRQ: St Georges Respiratory Questionnaire
BHQ: Bronchiectasis Health Questionnaire
CT: Computerised tomography
SD: Standard deviation
IQR: Interquartile range
CI: Confidence interval
