# Supplementary material for: The Objective Assessment of Cough Frequency in Bronchiectasis
Source: Lung. 2017 Jul 13;195(5):575–85. doi: 10.1007/s00408-017-0038-x (PMC5599483; doi:10.1007/s00408-017-0038-x)
Supplement: Supplementary file 1 — Supplementary material 1 (DOCX 11 kb) [file 408_2017_38_MOESM1_ESM.docx]

**ONLINE SUPPLEMENT FIGURE LEGENDS**

**Online resource 1** Relationship between 24-hour cough counts and cough severity Visual Analogue Scale (VAS). ρ: Spearman’s correlation coefficient. Objective cough counts per 24 hours were measured using the Leicester Cough Monitor.

**Online resource 2** Relationship between 24-hour cough counts and sputum Visual Analogue Scale (VAS). ρ: Spearman’s correlation coefficient. Objective cough counts per 24 hours were measured using the Leicester Cough Monitor.

**Online resource 3** Relationship between 24-hour cough counts and health status, using the St George’s Respiratory Questionnaire (SGRQ). r: Pearson’s correlation coefficient. Objective cough counts per 24 hours were measured using the Leicester Cough Monitor. Health status was measured using the St George’s Respiratory Questionnaire.

**Online resource 4** Relationship between 24-hour cough counts and Forced Expiratory Volume in the first second (FEV_1_). r: Pearson’s correlation coefficient. FEV1: Forced Expiratory Volume in the first second % predicted.

**Online resource 5** Relationship between 24-hour cough counts and Forced Vital Capacity (FVC). r: Pearson’s correlation coefficient. FVC: Forced Vital Capacity % predicted.
