# Supplementary figures and images for: The Objective Assessment of Cough Frequency in Bronchiectasis
Source: Lung. 2017 Jul 13;195(5):575–85. doi: 10.1007/s00408-017-0038-x (PMC5599483; doi:10.1007/s00408-017-0038-x)

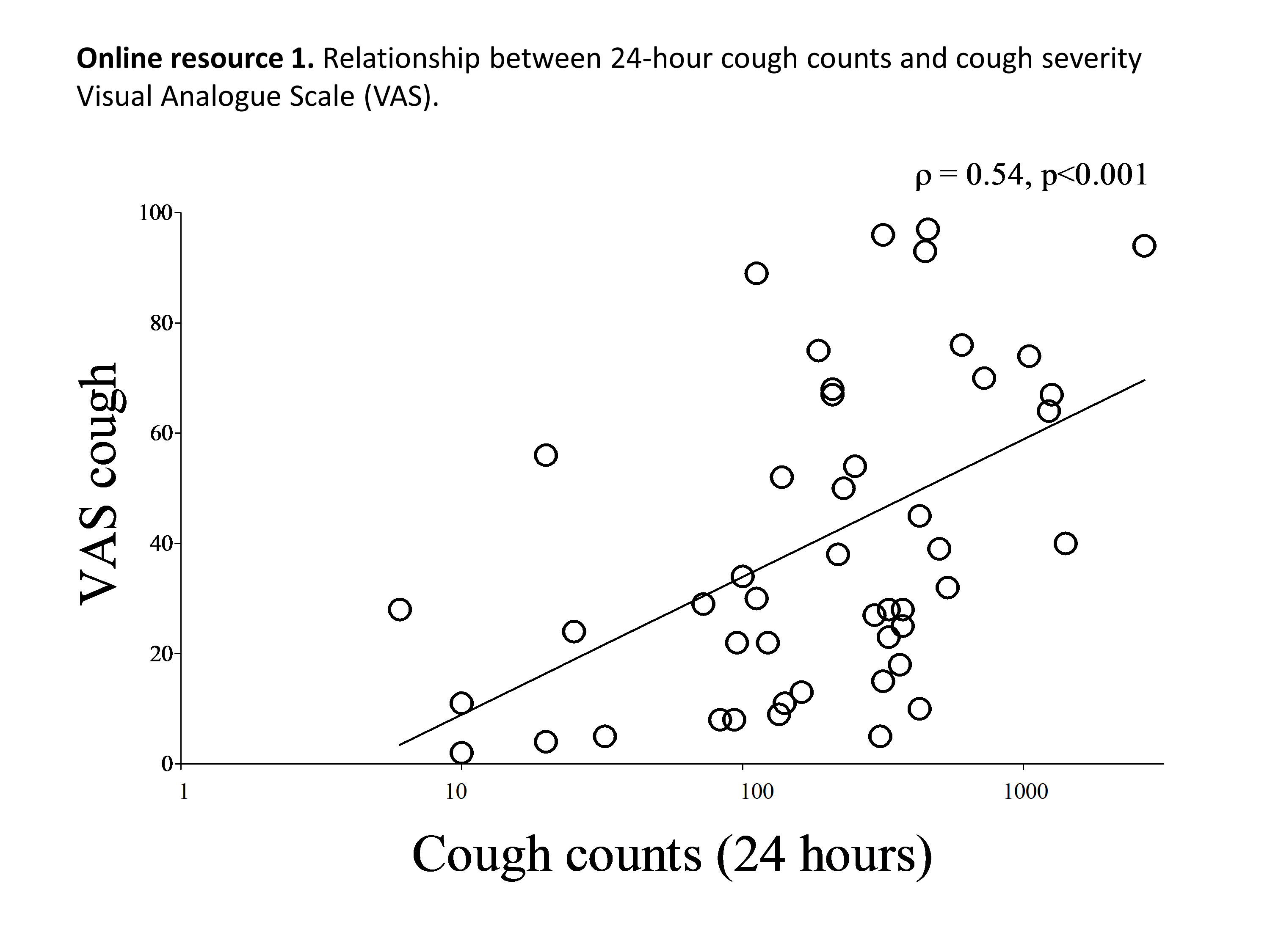

Supplement: Supplementary file 2 — Supplementary material 2 (TIFF 153 kb) [file 408_2017_38_MOESM2_ESM.tif]

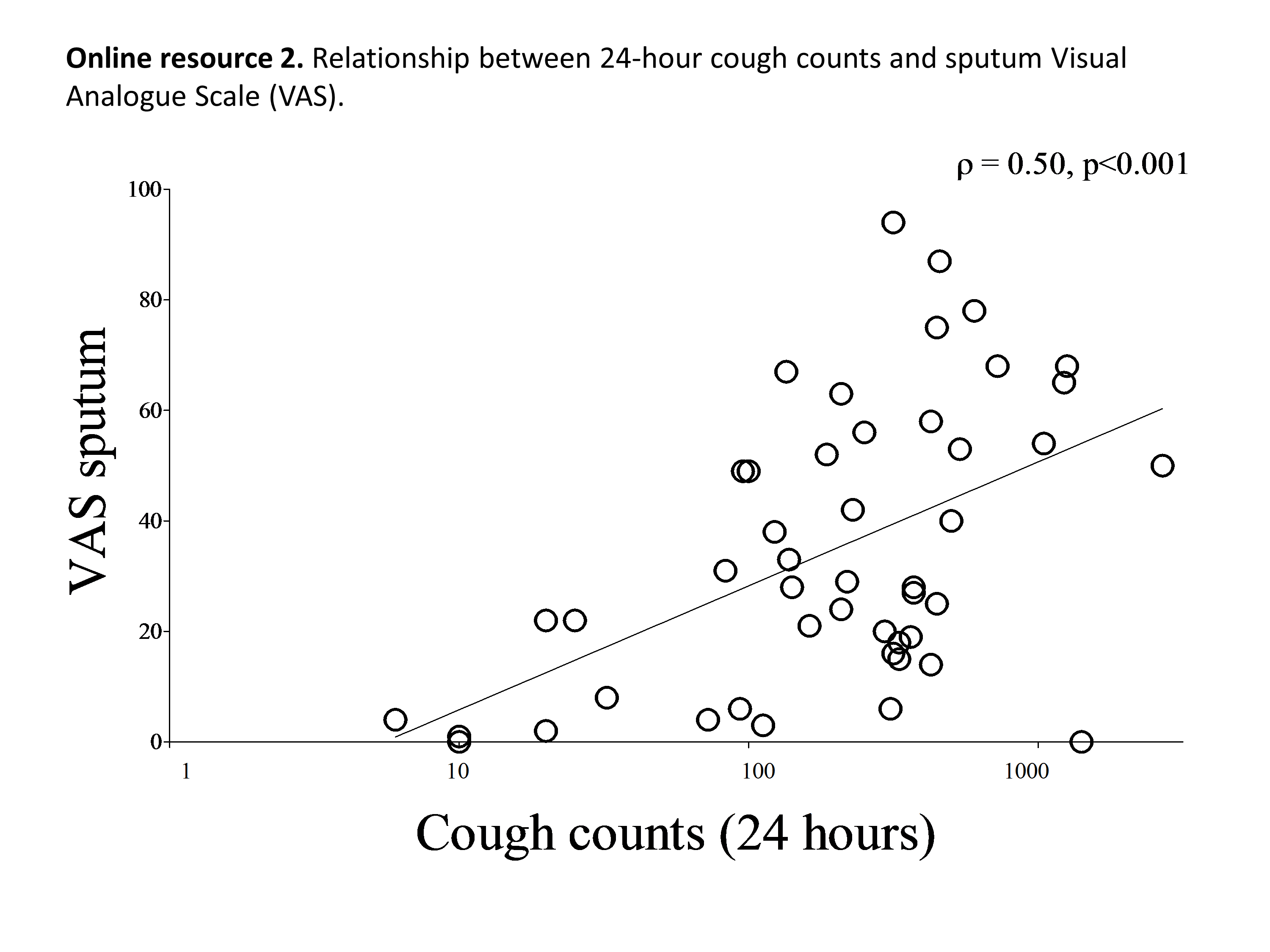

Supplement: Supplementary file 3 — Supplementary material 3 (TIFF 150 kb) [file 408_2017_38_MOESM3_ESM.tif]

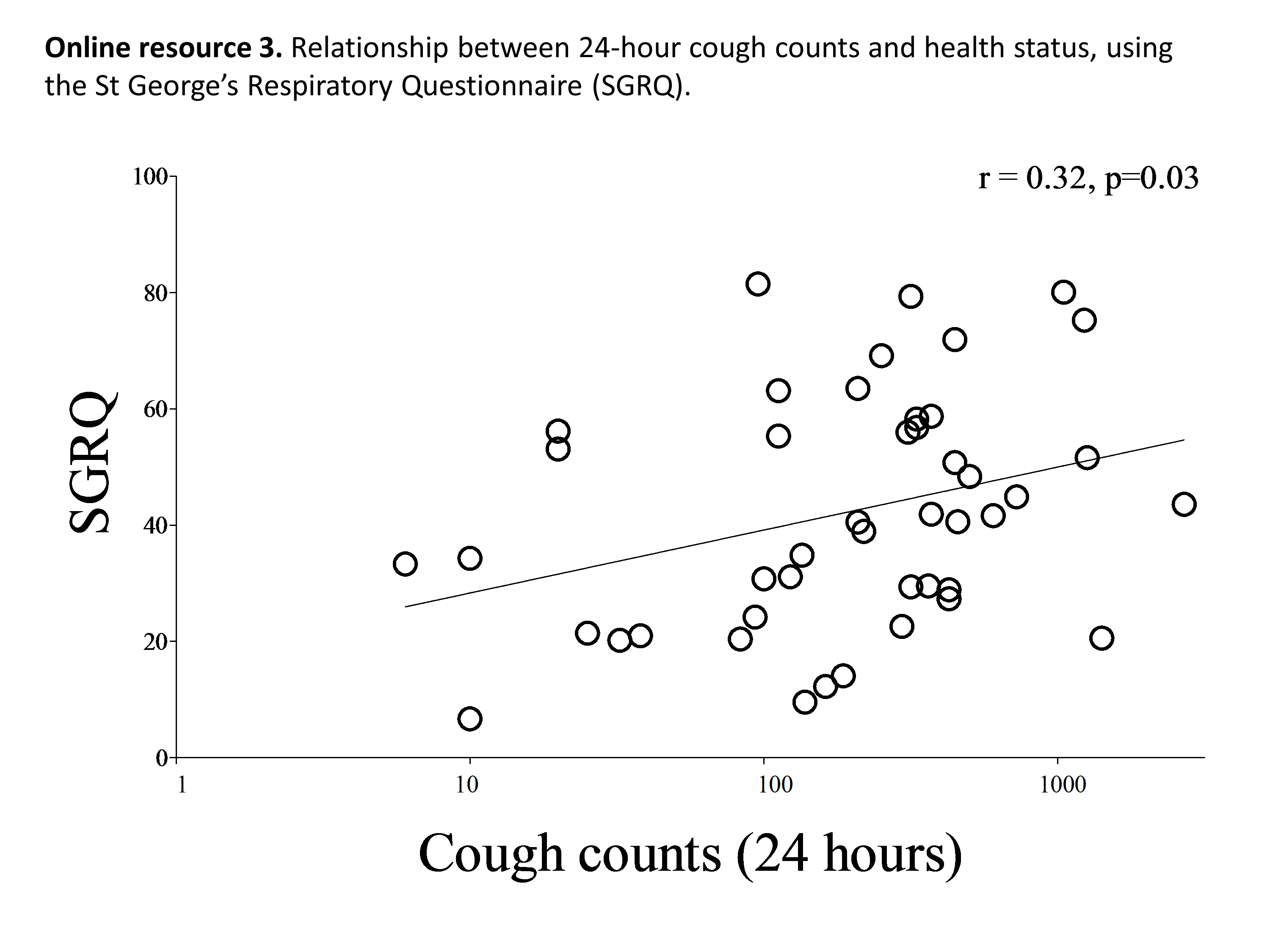

Supplement: Supplementary file 4 — Supplementary material 4 (TIFF 161 kb) [file 408_2017_38_MOESM4_ESM.tif]

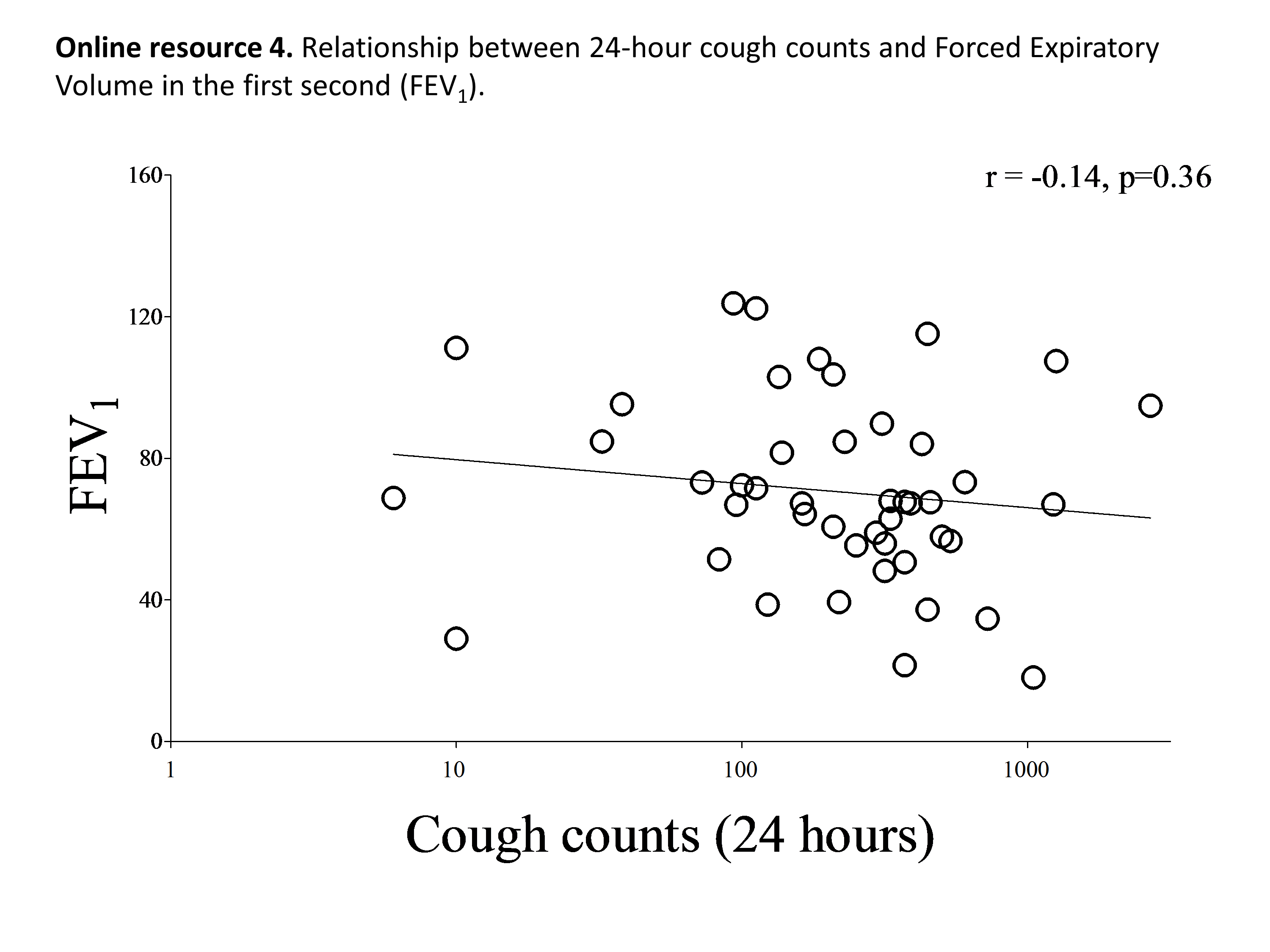

Supplement: Supplementary file 5 — Supplementary material 5 (TIFF 146 kb) [file 408_2017_38_MOESM5_ESM.tif]

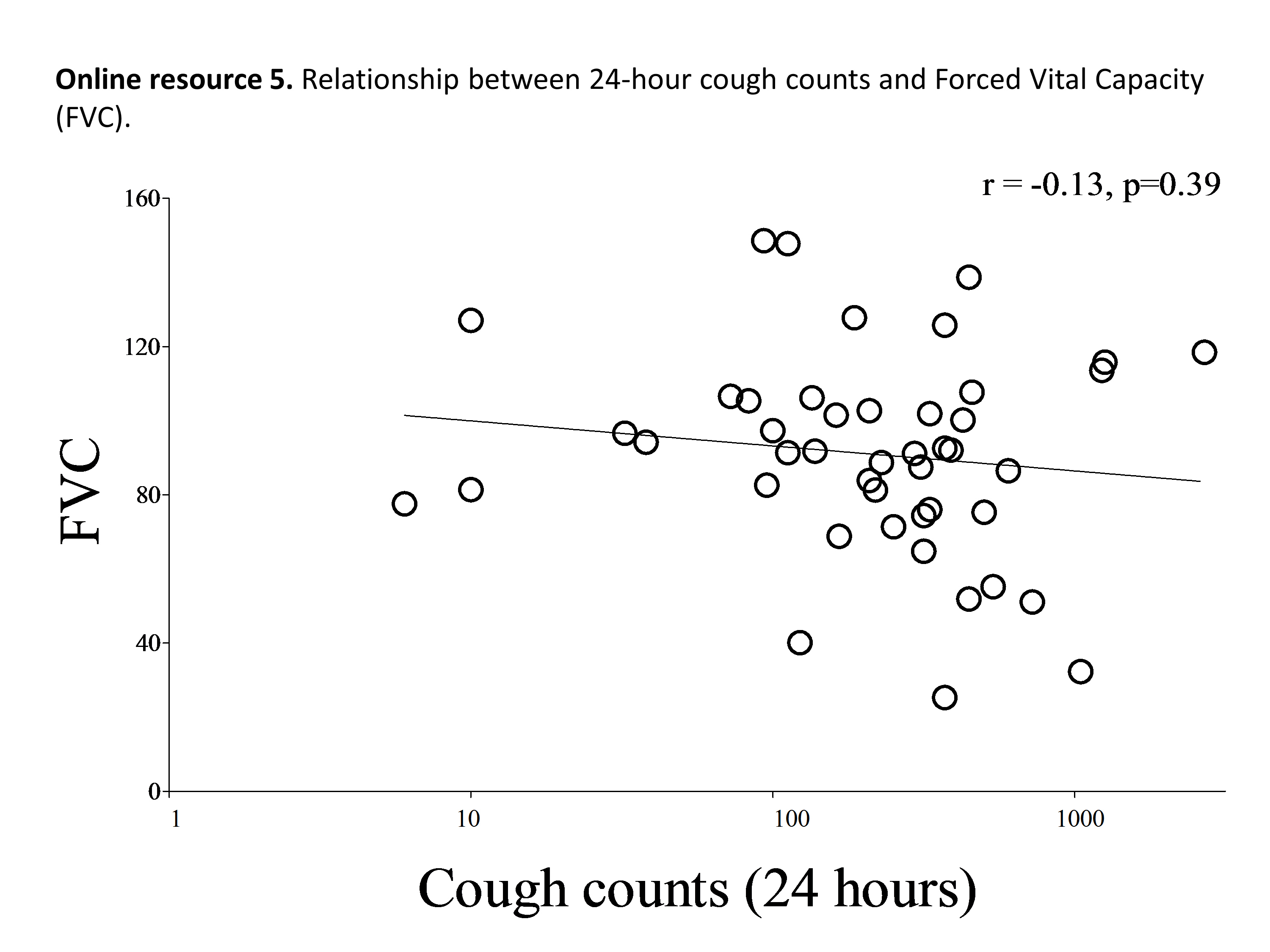

Supplement: Supplementary file 6 — Supplementary material 6 (TIFF 138 kb) [file 408_2017_38_MOESM6_ESM.tif]
